# Supplementary material for: Asymmetric cortical projections to striatal direct and indirect pathways distinctly control actions
Source: bioRxiv. 2025 May 3:2023.10.02.560589. Originally published 2023 Oct 2. Preprint. [Version 2] doi: 10.1101/2023.10.02.560589 (PMC10592949; doi:10.1101/2023.10.02.560589)
Supplement: Supplementary file 1 [file NIHPP2023.10.02.560589V2-supplement-1.pdf]

bioRxiv preprint doi: https://doi.org/10.1101/2023.10.02.560589; this version posted May 3, 2025. The copyright holder for this preprint (which was not certified by peer review) is the author/funder, who has granted bioRxiv a license to display the preprint in perpetuity. It is made available under aCC-BY 4.0 International license.

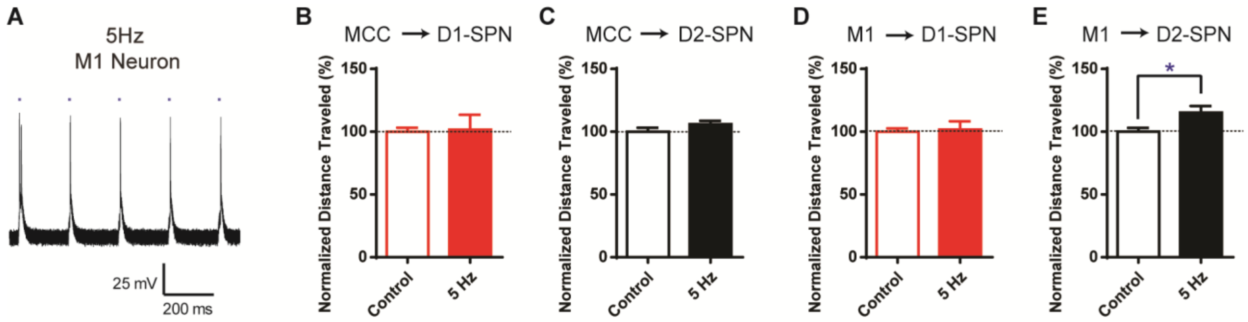

Figure S1. Low-frequency (5 Hz) optogenetic stimulation of cortical neurons projecting to striatal D1- or D2-SPNs has little effect on locomotion activity. (A) 5 Hz optogenetic stimulation elicits action potentials with high fidelity in a ChR2-mCherry positive M1 pyramidal neuron in layer 5 projecting to striatal D1-SPNs. Scale bars, 200 ms, 25 mV. **(B-C)** 5 Hz optogenetic stimulation on MCC neurons projecting to either D1- or D2-SPNs didn't change the locomotion activity. MCC – D1, n = 9, unpaired two-tailed t-test, t = 0.1906, P = 0.8516. MCC – D2, n = 10, unpaired two-tailed t-test, t = 1.015, P = 0.3275. **(D)** 5 Hz optogenetic stimulation of M1 neurons projecting to D1-SPNs didn't change the locomotion activity. n = 7, unpaired two-tailed t-test, t = 0.276, P = 0.7866. **(E)** 5 Hz optogenetic stimulation of M1 neurons projecting to D2-SPNs slightly increased locomotion activity. n = 8, unpaired two-tailed t-test, t = 2.48, P = 0.0265. *, $P < 0.05$.

bioRxiv preprint doi: https://doi.org/10.1101/2023.10.02.560589; this version posted May 3, 2025. The copyright holder for this preprint (which was not certified by peer review) is the author/funder, who has granted bioRxiv a license to display the preprint in perpetuity. It is made available under aCC-BY 4.0 International license.

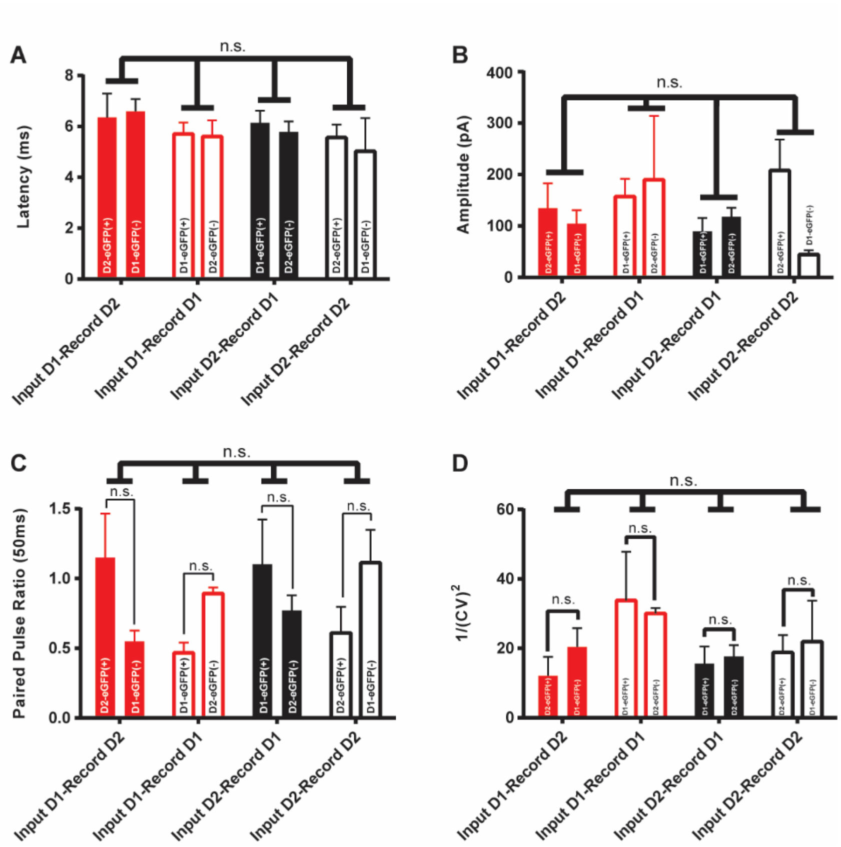

Figure S2. The synaptic properties of projections from D1- or D2-SPN retrogradely-labeled cortical inputs to striatal D1- or D2-SPNs. (A-D) The EPSC latency (A), amplitudes (B), paired pulse ratio (C) and variation (D) of whole-cell recordings of rabies-negative striatal D1- or D2-SPNs, with optogenetic stimulation of the terminals of D1- or D2-SPN retrogradely-labeled cortical neurons. n.s., $P > 0.05$, not statistically significant.

bioRxiv preprint doi: https://doi.org/10.1101/2023.10.02.560589; this version posted May 3, 2025. The copyright holder for this preprint (which was not certified by peer review) is the author/funder, who has granted bioRxiv a license to display the preprint in perpetuity. It is made available under aCC-BY 4.0 International license.

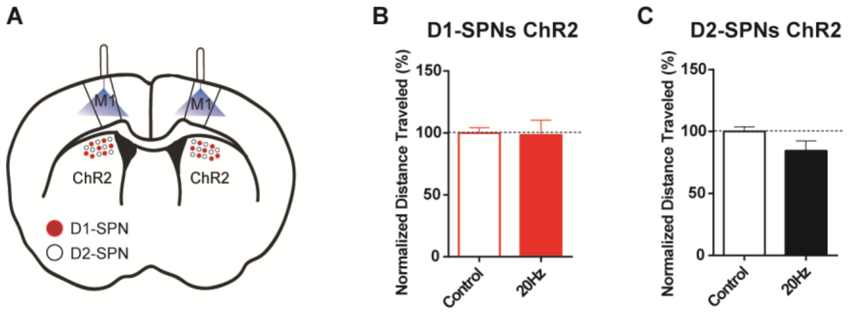

Figure S3. No effects of optogenetic stimulation of M1 on locomotion in mice with ChR2 expression in either D1- or D2-SPNs of DMS. (A) Schematic of dorsal medial striatum (DMS) injection of Cre-dependent AAV-ChR2 in D1- and A2a-Cre mice with optogenetic simulation in M1. **(B)** 20Hz optogenetic stimulation of M1 in mice expressing ChR2 in striatal D1-SPNs didn't change the locomotion activity. $n = 5$, unpaired two-tailed t-test, $t = 0.1016$, $P = 0.9194$. **(C)** 20Hz optogenetic stimulation of M1 in mice expressing ChR2 in striatal D2-SPNs didn't alter the locomotion activity. $n = 5$, unpaired two-tailed t-test, $t = 1.155$, $P = 0.2525$.
